# Healthy Moms and Babies Preventive Psychological Intervention Application: A Study Protocol

**DOI:** 10.3390/ijerph182312485

**Published:** 2021-11-27

**Authors:** Natalia Ruiz-Segovia, Maria Fe Rodriguez-Muñoz, Maria Eugenia Olivares, Nuria Izquierdo, Pluvio Coronado, Huynh-Nhu Le

**Affiliations:** 1 Department of Psychology, Universidad Nacional de Educación a Distancia (UNED), 28040 Madrid, Spain; nruiz252@alumno.uned.es; 2 Department of Gynecology and Obstetrics, Instituto de Salud de la Mujer José Botella Llusiá, Hospital Clínico San Carlos, Faculty of Medicine, Universidad Complutense de Madrid, 28040 Madrid, Spain; meolivares@cop.es (M.E.O.); nizquierdo@salud.madrid.org (N.I.); pluviojesus.coronado@salud.madrid.org (P.C.); 3 Department of Psychology, George Washington University, Washington, DC 20052, USA; hnle@gwu.edu

**Keywords:** perinatal depression, prevention, application

## Abstract

Depression is the most common psychological disorder during the perinatal period, and its negative effects extend to mothers, babies, their family and society. Scientific evidence points to the urgency of designing preventive interventions and concludes that the gestational period is the most appropriate time to implement these interventions. However, many pregnant women do not seek professional help due to a lack of knowledge about the importance of mental health, its impact, and the available intervention options, as well as a lack of time and financial resources. E-health interventions can be an efficient, cost-effective, and accessible resource for preventing postpartum depression that can circumvent the barriers that pregnant women face. This randomized clinical trial will examine the efficacy of Healthy Moms and Babies, an app aimed at preventing postpartum depressive symptomatology. The second objective of this study is to analyze the effectiveness of the tool in preventing anxious symptomatology. The primary outcome measure is the difference in the mean score between the intervention and control groups on the Patient Health Questionnaire-9 (PHQ-9) at the end of the intervention and at 3 and 6 months postpartum. The secondary outcome will be determined by using the Generalized Anxiety Disorder Screener (GAD-7) at the same time points. The research findings can be used to determine pregnant women’s use of the e-health application for the prevention of postpartum depression, whether the Healthy Moms and Babies intervention app is an effective and useful resource, and what modifications will need to be made to the tool in future updates.

## 1. Introduction

Depression is the most common psychological disorder during the perinatal period [1] and is a major public health problem that requires special attention [2,3]. Despite being a globally recognized and studied mental health condition, depression remains an underscreened and undertreated condition; much remains to be done for individuals with depression [4].

Perinatal depression is present worldwide. In the puerperal period, the prevalence of depression ranges from 4% to 63.9% depending on the country [5]. Rates of depression in industrialized countries range between 10% and 15%, but the rates are higher and increase to 20% in developing regions [6]. In Spain, the prevalence of moderate–severe depressive symptoms ranges between 14.8% [7] and 15.2% [8], and between 10.5% and 12.7%, during pregnancy and postpartum, respectively, depending on the time of assessment [9,10]. Furthermore, there is a high comorbidity between symptoms of perinatal depression and anxiety [11]. In Spain, the prevalence of anxiety ranges from 16.8% to 19.5% throughout pregnancy [12], and it is a strong predictor of postpartum depression [13].

The effects of perinatal depression extend beyond the mother, affecting the baby, partner, and family. Perinatal depression can disrupt caregiving activities [14] and the establishment of secure attachment [15]. Slomian et al. [16] categorized the negative outcomes of perinatal depression into three areas: (1) the mother’s physical and psychological health, with increased risk behaviors during the pre- and postnatal period (e.g., noncompliance with medical check-ups and unhealthy lifestyles); (2) the baby’s emotional, social, and behavioral development, with increased risk of lifelong psychopathology [17]; and (3) mother–baby interactions, including disruptions in breastfeeding and bonding.

Despite the negative consequences associated with perinatal depression, most women do not receive screening or treatment for perinatal depression although screening tools and evidence-based treatments are available [4]. Over 50% of women with antenatal or postpartum depression are unrecognized and undiagnosed, and few women (less than 20%) receive treatment [18]. Therefore, scientific institutions have stressed the need for interventions to prevent the consequences outlined above. In the United States (US), the Preventive Task Force [19] urges health professionals to refer women at risk for perinatal depression to counseling services. Developing successful psychological interventions to prevent perinatal depression improves parenting behaviors, it improves the quality of the mother–child relationship, and it enhances positive child outcomes such as better mother–child interactions or children’s mental health [20]. Moreover, the benefits of successful interventions appear to extend to the rest of society. The results of studies on the cost-effectiveness of preventive interventions for postpartum depression are still uncertain and inconclusive due to the methodological heterogeneity of the research [21], but the results are encouraging. The findings suggest that untreated perinatal depression may have important economic implications for the health care system. In the US, a mathematical model estimated that the societal cost of untreated perinatal mood and anxiety disorders from conception through 5 years postpartum was USD 14 billion for all births that occurred in 2017 [22], with more than half of the costs occurring during conception through birth. An Australian study suggests that if the prevalence rate of postpartum depression could be reduced by 5%, costs could be reduced by up to CAD 147 million [23].

As Muñoz et al. [3] point out, efforts should focus on preventing depression during pregnancy or the first weeks after childbirth for important reasons: (1) pregnancy and the twelve months after childbirth are a window of risk for the development of depressive symptomatology; (2) psychological interventions could be integrated in prenatal classes, which would reduce costs and associated stigma; and (3) the benefits of preventing depression extend to the baby even before birth. Pregnant women have frequent contacts with health services and are more willing to receive help because they believe it will have a positive impact on their baby [24]. However, there are barriers to seeking treatment and addressing prevention during this time. These barriers include the lack of information and knowledge about emotional problems and the available intervention options, as well as the practical limitations of access to treatment, such as the economic and time costs [25], which are closely related to putting the care of the child before the mother’s own care. Mental health illiteracy, myths and misconceptions promote prejudices about postpartum depression that are related to feelings of weakness and the belief of being a “bad mother”, which are other prominent barriers to seeking help [26]. Due to the stigmatization of mental health, especially in the perinatal period, women are reluctant to go to specialized units and prefer to receive care in obstetric and gynecological centers that are separated from mental health units [27]. Additionally, it is still difficult to find multidisciplinary teams in obstetric departments with a designated psychologist, and many women report the unwillingness of health professionals to attend to and support their emotional needs [28].

Given the advances in information and communication technologies, e-health interventions can help to overcome the aforementioned barriers, as these interventions preserve anonymity, are flexible and accessible [29], and can contribute to emotional and psychological literacy, making it easier for anyone to obtain information and help [3]. Systematic reviews on the effectiveness of e-health interventions for the prevention and reduction of depressive symptomatology in the perinatal period have concluded that such interventions can improve mothers’ moods [30] and reduce depressive symptoms [31].

Computer and internet-based e-health interventions have been developed and tested, but few studies have examined these interventions [29]. Danaher et al. [32] developed a web-based intervention program for postpartum depression called MomMoodBooster. The intervention program is comprised of six self-guided sessions, and it includes a weekly call from a therapist. It offers the ability to personalize the experience by uploading photos and setting goals and provides access to a private web forum for mothers to interact with their peers. The results indicated a statistically significant decrease in depressive symptomatology at the end of the program and at the six-month follow-up [32]. Fonseca and colleagues developed the Be a Mom program [33,34], which is a self-guided web-based intervention for postpartum women to prevent persistent postpartum depressive symptoms. The program is based on the principles of cognitive behavioral therapy and incorporates recent approaches based on third-generation therapies. The results suggested that the program promotes women’s self-compassion and improves their emotional regulation abilities, skills that exert a protective effect in the presence of risk factors for postpartum depression. Barrera et al. [35] adapted the Mothers and Babies Course to an online platform to prevent perinatal depression. Their results indicate that the benefits of participating in this online intervention were larger for pregnant women reporting high levels of prenatal depression symptoms. Furthermore, Duffecy et al. [36] developed a cognitive behavioral therapy internet intervention combined with peer support. The results suggested that women were responsive to both aspects to prevent perinatal depression. It is important to note that all the aforementioned interventions focused mostly on the psychological aspects (i.e., perinatal depression) without addressing other relevant content, such as breastfeeding, nutrition or the birth process.

In the field of e-health, mobile applications represent a tool with great potential in the gestational and postpartum periods. A study by Osma et al. [37] concluded that pregnant women use the internet more frequently from their smartphones than from their computers, with the mobile phone being the digital device that is most used by this population. Most women use pregnancy and parenting-related applications [38,39], and even women with few economic resources make use of these applications [40]. Pregnant women use applications for information when conventional antenatal care resources do not meet their needs or when they experience difficulty accessing these resources [41]. In addition, these applications allow for screening, assessment, and psychological interventions to be performed anywhere, anytime and at a low cost [42], increasing the chances of early detection and of providing rapid and appropriate interventions [43]. A meta-analysis of mobile application interventions for mental health confirmed their efficacy, highlighting their potential as cost-effective and accessible interventions for women who are unable to receive standard psychological treatment [44], and showed that these interventions offer promising results in improving and maintaining health. Notably, most of the research on perinatal depression prevention with e-health has been carried out in English-speaking countries [45] (e.g., the United States and Australia), while studies in Spanish are limited to Barrera and her colleagues. During 2019 [46], Spain reported 377,906 single births, 8134 double births, and 93 triple births or more; therefore, the number of women suffering from postpartum depression could range from 42,643 to 104,782. An application designed in the Spanish language will be very helpful to address the needs of the many women who could suffer depression all over the country.

The primary objective of the current study is to determine whether a Spanish preventive psychological intervention app designed in Spain, Healthy Moms and Babies, is superior to the general delivery of pregnancy information (breastfeeding advice, nutrition and exercise, birth facts, fetal development information and answers to frequently asked questions) in the prevention of postpartum depressive symptomatology. The secondary objective is to examine whether this same intervention is more effective than the general delivery of information about pregnancy in preventing postpartum anxious symptomatology.

## 2. Methods

### 2.1. Design and Population

**Figure 1:**
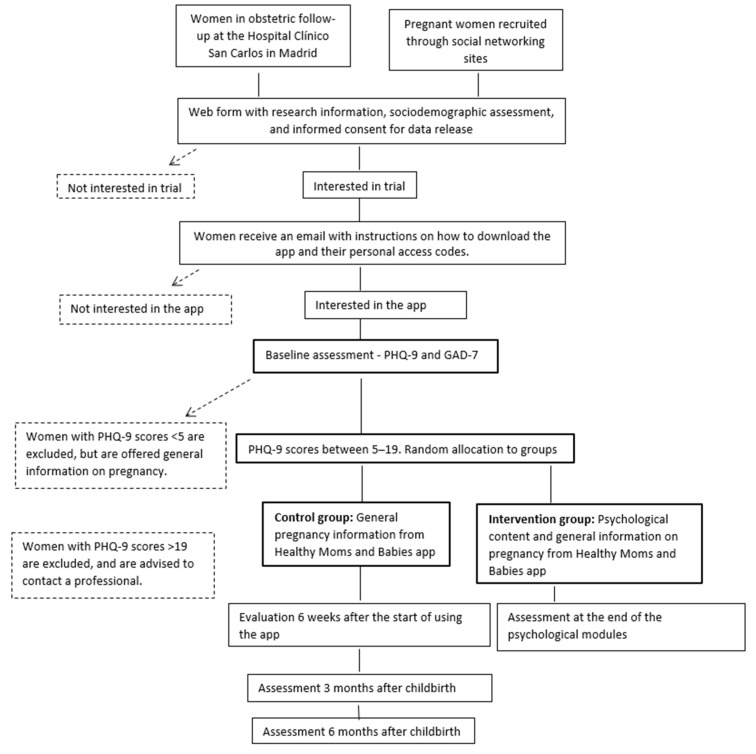
Flowchart of the study design.

The design is a two-armed randomized controlled study, with patients assigned to the intervention group, who will have access to the psychological content of the Healthy Moms and Babies app and general information about pregnancy, while those assigned to the control group, who will only have access to the informational content provided by the e-health tool. Participants will be assessed before and after using the app and at three and six months after their due date. Figure 1 shows a flow chart of the study design. Recruitment will take place between 2020 and 2022.

### 2.2. Recruitment of Women

#### 2.2.1. Identification and Recruitment in the Obstetrics and Gynecology Department

The obstetricians and midwives who collaborate in the research will invite pregnant women who are undergoing obstetric follow-up at the Hospital Clínico San Carlos in Madrid to participate in the study. Interested individuals will be given a leaflet explaining the research with a QR code that they can scan with their smartphone to visit the initial web form that collects contact and socio-demographic data (age, gestational week, place of origin and residence, reference hospital, completed studies, employment status, marital status, and number of pregnancies, cesarean sections and/or previous abortions).

#### 2.2.2. Mass Recruitment on Social Media

In parallel to recruitment at the hospital, announcements and ads will be published on the social networks, Twitter and LinkedIn, encouraging pregnant women from the general population to participate in the research study. Women will be provided with a direct link to the website of the Healthy Moms and Babies project (https://blogs.uned.es/mbc/, accessed on 26 November 2021), which, in turn, redirects them to the initial web form.

#### 2.2.3. Evaluation, Downloading Instructions and Accessing Passwords

Participants who complete the initial form will be asked to provide informed consent. Following this, women will receive a welcome letter and thank you email for their participation with instructions for downloading the Healthy Moms and Babies application and their personal access codes.

### 2.3. Outcomes and the Allocation of Participants to Trial Groups

#### 2.3.1. Primary Outcome

The primary outcome measure will be the difference in the mean scores between the intervention and control groups on the Patient Health Questionnaire-9 (PHQ-9) at the end of the psychological modules of the Healthy Moms and Babies application (in the case of the control group, the post assessment will be done six weeks after starting the PPP for the first time) and at three and six months after the delivery date.

The PHQ-9 is a nine-item instrument that assesses the severity of depressive symptomatology according to the following criteria of the Diagnostic and Statistical Manual of Mental Disorders [47]: depressed mood, markedly diminished interest in usual activities, significant increase or loss of appetite/weight, insomnia/hypersomnia, psychomotor agitation/retardation, fatigue or loss of energy, feelings of worthlessness or guilt, difficulty with thinking, concentrating or making decisions, and recurrent thoughts of death or suicide. The items are rated on four-point Likert-type scales, ranging from 0 (never) to 3 (almost every day), with higher scores indicating high severity of depressive symptoms (range 0–27). A score of 10 is the cut-off point recommended in clinical practice for estimating the presence of depressive symptoms [48,49]. However, considering that our aim is to prevent depressive symptoms, we will include all pregnant women who obtain a score equal to or greater than 5. The PHQ-9 has been validated in Spanish-speaking samples and in Spanish pregnant women [50].

#### 2.3.2. Secondary Outcome

The secondary outcome measure will be the difference in the mean scores between groups on the Generalized Anxiety Disorder Screener (GAD-7) at the same time points described above.

Anxiety symptoms will be assessed using the GAD-7, which is a self-administered instrument based on the criteria of the Diagnostic and Statistical Manual of Mental Disorders [47]. The total score ranges from 0 to 21, with the highest scores being the most severe [51]. The GAD-7 has been validated for use in Spanish-speaking pregnant women [52].

### 2.4. Inclusion and Exclusion Criteria

Inclusion criteria include being pregnant (participants can join the study at any time during their pregnancy), being over 18 years of age, understanding and speaking Spanish fluently, having a smartphone and the basic skills to operate it, and giving consent to participate in the study. The exclusion criteria for the study are not being pregnant, not understanding or speaking Spanish fluently and not having a smartphone or the basic skills to use it.

### 2.5. Randomization

When participants access the Healthy Moms and Babies application for the first time, they will complete the baseline assessment including the PHQ-9 and GAD-7. Only participants who score between 5 and 19 on the PHQ-9 will be included and randomized into the treatment and control groups.

Based on the PHQ-9 score, called the $value, the Plesk software will distribute participants according to their level of symptomatology. Participants scoring <5 on the PHQ-9 have minimal depressive symptoms and therefore they will be excluded from the study and no further evaluation will be performed, but they will be offered access to the pregnancy information content. Participants scoring >19 on the PHQ-9 are considered to have severe symptomatology and will also be excluded from the study. In this case, these participants will be blocked from accessing the Healthy Moms and Babies application because it was not designed to address severe depressive symptomatology. Women will receive an alert message telling them to share their distress with someone close to them and to see a specialist for appropriate psychological care. Eligible patients (PHQ-9 score between 5 and 19) will be randomly assigned to the intervention and control groups according to the standard PHP function (mt_rand()) automatically generated by a computer. Women in the intervention group will have access to the psychological content modules and the general pregnancy information on the Healthy Moms and Babies app; women in the control group will only be able to access the informational content.

An independent researcher will program the mathematical algorithm that performs the randomization. Subsequently, simple blinding will be used, and participants will not be informed of the group to which they are assigned. The allocation will be known to the study team after it is performed automatically.

Outcome measures will be assessed at baseline, immediately after the intervention, and three and six months after the delivery date. At follow-up, questionnaires will be sent at the appropriate time by email and will be completed by the women via a web form.

### 2.6. Adherence

Participants in the intervention group will be asked to complete activities at the end of each psychological content module and will be invited to put into practice what they had learned during the week. To increase the women’s motivation and improve adherence, while using the Healthy Moms and Babies app, women will be randomly asked true/false quiz questions about the content that is already covered in the modules. There is no compensation to participate in the study.

### 2.7. Ethical Standards

This study is conducted according to the principles expressed in the Declaration of Helsinki. This study has been approved by the Institutional Review Board at the San Carlos Clinic Hospital (Reference number 19/184-E). Handling of the study data complies with all the Spanish required standards for data protection. Electronic informed consent will be obtained from all the participants, and the confidentiality of all the provided information will be ensured.

### 2.8. Sample Size

A representative sample size was calculated according to the number of newborns in the previous year in each country. Thus, we estimated a minimum sample size of 385 participants, based on an α-level of 0.05 and heterogeneity equal to 50%.

### 2.9. Analysis Plan

The primary analysis will compare the mean score on the PHQ-9 between the two trial groups before and after the intervention and at three and six months postpartum to assess the effectiveness of the Healthy Moms and Babies app in preventing postpartum depressive symptomatology. Similar analyses will be conducted for the secondary outcome on anxious symptomatology. In addition, a qualitative study of the participants’ responses to the developmental exercises proposed in some modules will be conducted. The severity of depressive and anxious symptomatology will be studied in relation to the sociodemographic characteristics that were assessed, and the participants’ use of the application will be examined (number of visits to the app, most visited content, time invested in completing the modules, the results of the quiz questions, etc.).

## 3. Intervention

### 3.1. Rationale

The intervention was based on the Mothers and Babies Course [53] for the prevention of postpartum depression. This intervention has demonstrated strong empirical evidence for preventing perinatal depression [19].

The Mothers and Babies Course is based on the principles of cognitive behavioral therapy (CBT) and attachment theory and includes three sections that address the main components of CBT: thoughts, pleasant activities, social contacts. Cognitive behavioral therapy is one of the most studied interventions for the treatment and prevention of postpartum depression. The Mothers and Babies Course uses a healthy management of reality model that helps women recognize how their internal and external realities can affect their mood, and how to manage their thoughts, behaviors, and relationships to promote healthy emotional states and reduce the risk for depression [3]. Elements of attachment therapy are also integrated into the app with guidelines for developing a secure attachment with their baby and promoting the connection between the mother and the positive impact it has on her child. In addition, the Healthy Moms and Babies application includes content on problem solving and decision-making, relaxation training, and demystifying misconceptions and biases about motherhood.

Cognitive behavioral therapy appears to be the most empirically supported therapeutic approach [54,55]. Evidence indicates that CBT interventions are efficient and cost-effective in treating and preventing perinatal depression [54,56], and reducing symptoms and the progression of postpartum depression [55].

A multidisciplinary team of psychologists and obstetricians developed the content of the app by discussing and reflecting on the important topics to be included. First, the existing literature on preventive interventions for postpartum depression was reviewed, and the intervention’s content and activities were analyzed. Then, e-health interventions were examined to delve into their structure, function and other interesting elements in the online intervention. Based on the framework of the Mothers and Babies Course [53] and other literature reviews, one part of the team of professionals drafted the content script, and the other part performed a subsequent check, which included making appropriate modifications.

### 3.2. Goals of the Intervention

The primary objective of the e-health intervention application Healthy Moms and Babies was the prevention of depressive symptomatology during the postpartum period. The secondary objective was to prevent anxious symptoms after childbirth. Additionally, the application aims to raise awareness among pregnant women about the importance of taking care of their mental health and psychological well-being and the positive effects of taking care of their own well-being on their baby’s well-being.

### 3.3. Overview of the Intervention

The psychological intervention was comprised of eleven modules with a similar structure: an introductory video, psychoeducational content, a practical exercise, general guidelines, and motivational messages. Considering that a new module was released every 4 days, the average duration of the intervention was six weeks. The components of the intervention are described in the following paragraphs and Table 1.

*Module 1. You are a mum:* welcomes, collects and validates all the emotions and sensations that a pregnant woman may experience, inviting her to make use of the app to live consciously during her pregnancy. The practical activity allows the participant to reflect on her emotions related to her pregnancy.

*Module 2. Psychoeducation:* validates any emotions related to pregnancy and motherhood, reducing guilt, and encouraging responsible action. It provides some facts and information about the baby blues, perinatal depression, and its consequences. The practical activity consists of pointing out the depressive symptoms with which the woman identifies and naming the situations in which she does so.

*Module 3. Feel better:* presents the story of Violeta and María, two women who encounter the same day, but with very different experiences. It addresses the individual responsibility of making choices to increase the feeling of well-being. The practical activity invites the participant to create positive loops of activities, thoughts, and emotions to help her through difficult moments.

*Module 4.Thoughts:* proposes that the participant listens to her thoughts and identifies the emotions she feels with each thought, inviting her to question them, release them or reformulate them when they generate discomfort and do not conform to reality. In the practical portion, the app challenges the woman to transform her most negative and harmful thoughts into healthier ones.

*Module 5.Enjoy the day:* addresses the importance of planning and prioritizing pleasant activities each day, highlighting their potential benefits for emotional health. It proposes that the participant reflects on five pleasant activities that can be done in their daily life and offers guidelines for time management and how to practice these guidelines daily.

*Module 6.* Relax: relaxation is presented as a deactivation technique in stressful situations. The app offers women five relaxation audio clips and invites the women to practice them during pregnancy and after childbirth with their baby.

*Module 7.Your body:* this module is the only one that does not propose a practical or reflective activity, and its content is basically informational. It provides information on the most common bodily and physiological changes during pregnancy.

*Module 8.Find a solution*: differentiate between proactive and reactive tasks or emotion-focused coping strategies. The app proposes that the participant reflects on the aspects of her life that she wants to improve and invites her to look for alternative coping techniques to those she has used thus far.

*Module 9.Social support*: highlights the importance of social support and differentiates between the different types. It also offers guidelines for assertive communication in the active search for support. This module suggests that the participant writes down a list of people who can provide support and suggests that she reflects on her support needs during pregnancy and the postpartum period.

*Module 10.Goodbye myths*: uncovers the preconceived misconceptions about motherhood that can lead to frustration and suffering.

*Module 11.Your baby*: addresses the participant’s expectations of what her baby will be like. It also offers some general guidelines for developing a secure attachment bond and some specific tips for handling very demanding babies.

### 3.4. Email Reminders to Increase App Utilization

When the app detects that the participant has not progressed in the modules for several days or weeks, it will send an email reminder to the mother to: (a) encourage continuing participation with the app, (b) inform the participant of subsequent topics; (c) offer the opportunity for the participant to ask questions or request support in the use of the tool; and (d) provide instructions to activate notifications as to when the next psychological module will be unlocked. If the participant continues to not use the application, up to three emails will be sent encouraging her to do so; following this, the participant is considered lost to follow-up.

### 3.5. Intervention Quality Control Procedures

The Healthy Moms and Babies application was developed by a research team comprised of professionals in the field of health psychology and obstetrics. Email contact with the participants will be carried out by a psychologist from the team who is trained to respond to any clinical/crisis needs that may arise.

### 3.6. Usual Care

The Moms and Babies application does not interfere with the usual care that the pregnant women receives and is not incompatible with any other type of psychological intervention. Women who score <5 on the PHQ will be excluded from the study, and will be offered access to the general content of the application and information on breastfeeding, nutrition, and physical activity.

## 4. Discussion

Perinatal depression can threaten the physical and psychological health of the mother, put the baby’s development at risk, and affect the creation of the bond between mother and baby, the couple’s relationship, and the family [16]. To avoid these negative consequences, health providers [34] and institutions [19] must stress the need to implement preventive interventions for perinatal depression. Pregnancy is a favorable time to address prevention [3,24]. However, help-seeking rates during this period are low due to multiple barriers and difficulties, including the lack of information, costs of access to conventional treatments, and fear of stigmatization and public exposure to peers [33,34].

E-health interventions, including mobile applications, offer advantages in overcoming the barriers described above, facilitating access to interventions at low costs and in a simple way. Moreover, these applications can reach women all over the world, including those women who experience difficulty traveling to receive health services [57,58]. Digital tools offer new and tailored content, allowing women to access the tools at their own pace and preserving their anonymity [59].

However, despite the many virtues of online interventions [60], they also have some limitations. Participation and retention can be difficult to sustain in an online intervention, leading to high drop-out rates and low adherence [29]. It is therefore, important to consider the additional moderation of the intervention by a professional, analyze women’s use of the tool, and adapt the application to their needs as much as possible [61].

Mental health applications are not intended to replace professional clinical services. Instead, these apps can be a cost-effective, easily accessible, low-intensity resource for women who are unable to receive standard psychological treatment, or a good complementary resource to face-to-face counseling. The Healthy Moms and Babies app was developed by a multidisciplinary team that aims to contribute to increasing the scientific evidence on the effectiveness of CBT-based mobile applications to prevent postpartum depression.

## 5. Conclusions

This study contributes to efforts to make depression prevention a priority [37]. From a public health perspective, there is a need to address maternal mental health and technology-based resources can serve as a tool to help pregnant women and new mothers cope with perinatal depression symptoms

## Figures and Tables

**Table 1:** The components of the intervention.

Welcome	Content Always Available	General Information
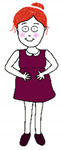	Info about the Healthy Moms and Babies app FAQs about pregnancy and childbirth Infant development Videos on childbirth Configuration of the app	Breastfeeding Feeding Physical activity
**Psychological Content**		
**Module 1**	**Module 2**	**Module 3**
**You are a mum**	**Psychoeducation**	**Feel better**
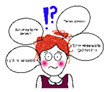 Collects and validates your emotions. Invites you to use the app.	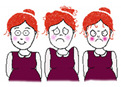 Info on PPD and its symptoms.	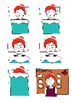 Well-being, enhancing decision-making.
**Module 4**	**Module 5**	**Module 6**
**Thoughts**	**Enjoy the day**	**Relax**
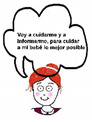 Cognitive restructuring, adjusting to reality, defusion strategies.	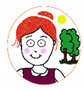 Behavioral activation with enjoyable activities. Time management and planning.	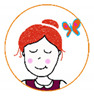 Relaxation audio clips for deactivation in stressful situations.
**Module 7**	**Module 8**	**Module 9**
**Your body**	**Find a solution**	**Social support**
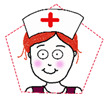 Info on body and physiological changes in pregnancy.	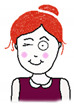 Coping and problem-solving strategies.	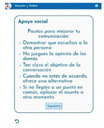 Info on social support, assertive communication guidelines and support-seeking.
**Module 10**	**Module 11**	
**Goodbye myths**	**Your baby**	
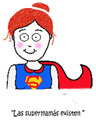 Myths about motherhood and their demystification.	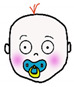 Addresses the woman’s expectations of the baby and guidelines for secure attachment development.	

## Data Availability

Not applicable.
